# High Mortality of Red Sea Zooplankton under Ambient Solar Radiation

**DOI:** 10.1371/journal.pone.0108778

**Published:** 2014-10-13

**Authors:** Ali M. Al-Aidaroos, Mohsen M. O. El-Sherbiny, Sathianeson Satheesh, Gopikrishna Mantha, Susana Agustī, Beatriz Carreja, Carlos M. Duarte

**Affiliations:** 1 Department of Marine Biology, Faculty of Marine Sciences, King Abdulaziz University, Jeddah, Saudi Arabia; 2 Marine Science Department, Faculty of Science, Suez Canal University, Ismailia, Egypt; 3 The UWA Oceans Institute and School of Plant Biology, University of Western Australia, Crawley, WA, Australia; 4 Department of Global Change Research and LINC Global, IMEDEA (CSIC-UIB) Instituto Mediterráneo de Estudios Avanzados, Miquel Marqués, Esporles, Spain; University of Vigo, Spain

## Abstract

High solar radiation along with extreme transparency leads to high penetration of solar radiation in the Red Sea, potentially harmful to biota inhabiting the upper water column, including zooplankton. Here we show, based on experimental assessments of solar radiation dose-mortality curves on eight common taxa, the mortality of zooplankton in the oligotrophic waters of the Red Sea to increase steeply with ambient levels of solar radiation in the Red Sea. Responses curves linking solar radiation doses with zooplankton mortality were evaluated by exposing organisms, enclosed in quartz bottles, allowing all the wavelengths of solar radiation to penetrate, to five different levels of ambient solar radiation (100%, 21.6%, 7.2%, 3.2% and 0% of solar radiation). The maximum mortality rates under ambient solar radiation levels averaged (±standard error of the mean, SEM) 18.4±5.8% h^−1^, five-fold greater than the average mortality in the dark for the eight taxa tested. The UV-B radiation required for mortality rates to reach ½of maximum values averaged (±SEM) 12±5.6 h^−1^% of incident UVB radiation, equivalent to the UV-B dose at 19.2±2.7 m depth in open coastal Red Sea waters. These results confirm that Red Sea zooplankton are highly vulnerable to ambient solar radiation, as a consequence of the combination of high incident radiation and high water transparency allowing deep penetration of damaging UV-B radiation. These results provide evidence of the significance of ambient solar radiation levels as a stressor of marine zooplankton communities in tropical, oligotrophic waters. Because the oligotrophic ocean extends across 70% of the ocean surface, solar radiation can be a globally-significant stressor for the ocean ecosystem, by constraining zooplankton use of the upper levels of the water column and, therefore, the efficiency of food transfer up the food web in the oligotrophic ocean.

## Introduction

Zooplankton are essential components of the marine food web, relaying primary production from microbial algae to fish and seabirds [1]. Pressures disrupting zooplankton communities can, therefore, generate considerable impacts on the marine food web and, particularly, reduce the flow of primary production upwards in the food web. Hence, there have been significant efforts at examining the impacts of global stressors, such as ocean warming [2], ocean acidification [3] and pollutants [4] on marine zooplankton.

UV-B radiation is emerging as a largely overlooked but prevalent stressor in the marine environment [5], [6], with increasing UV-B causing an increase in mortality across the taxa inhabiting the mixed layer of the ocean [6]. Concerns on increasing exposure of marine organisms to UV-B radiation derive from consideration of the combined effects of a global increase of the UV-B radiation incident in the ocean and increased penetration into ocean waters. The UV-B incident on the Earth surface has increased with the depletion of the stratospheric ozone layer four decades ago, with this increase being particularly strong over Antarctica but of global reach [7], [8]. Ozone values decreased, on average, 10.97±2% in the Southern Hemisphere compared to 2.72±0.45% in the Northern Hemisphere between 1970 and 2012 [8]. Evidence for increased UV-B penetration derives from indications, still awaiting confirmation, that the oligotrophic gyres, with the most transparent waters to UV-B, may be expanding in size [9] and that chlorophyll *a* concentration has declined globally at rates of about 1% per year, related to increasing sea surface temperature and vertical stratification [10]. Indeed, impacts of UV-B radiation are greatest in oligotrophic, transparent seas. For instance UV-B levels sufficient to cause significant mortality of photosynthetic plankton have been reported to penetrate down to 60 m in the oligotrophic waters of the subtropical Atlantic Ocean [11] and to 26 m in the Mediterranean Sea [12]. Yet, there is still limited understanding of the vulnerability of zooplankton to solar and UV-B radiation, as most studies have been conducted in alpine lakes and north-temperate coastal waters.

A recent meta-analysis demonstrated that crustaceans, a dominant component of zooplankton communities, are highly vulnerable to UV-B radiation [6]. Previous studies on planktonic (often neustonic) crustacean zooplankton showed that exposure to UV-B lead to behavioral [13] and physiological responses [14]–[16] ultimately affecting a range of fitness components [17] conducive to increased mortality [18]–[20]. Impacts of UV-B radiation is expected to be highest in tropical oligotrophic marine regions [11], where high incident UV-B radiation combines with deep penetration to yield high UV-B doses to organisms inhabiting the mixed layer. Yet, the functional responses of zooplankton mortality to ambient solar radiation doses have been assessed only in productive, turbid coastal waters [14]–[20]. Here we show, based on experimental assessments of solar radiation dose-mortality curves on eight common taxa, the mortality of zooplankton in the oligotrophic waters of the Red Sea to increase steeply with ambient levels of solar radiation.

The Red Sea is particularly prone to high solar and UV-B radiation in the mixed layer because (1) this region receives very high solar and UV radiation, with UV indices in excess of 12, in spring [21], [22]; (2) it is one of the most oligotrophic seas in the world, leading to high UV-B penetration reported to damage phytoplankton communities in the Gulf of Aqaba, Northern Red Sea [23]; and (3) the mixed layer is much shallower, at around 30 to 45 m depth [24], [25], than typically observed in the open oligotrophic ocean, implying exposure to very high solar and UV-B doses for organisms inhabiting the mixed layer.

## Materials and Methods

The sensitivity of the main zooplankton taxa in the Red Sea coastal ecosystem to solar radiation was assessed through experiments examining the dose-response curves relating mortality rates of zooplankton taxa to solar radiation. In short, the experiments involved exposing quartz bottles containing a number of individuals to five levels of solar radiation allowing100%, 21.6%, 7.2%, 3.2% and 0%, and assessing the mortality in the quartz bottles at regular time intervals until 90% of mortality was obtained. The mortality rates and cumulative doses received at the various treatments allowed calculation of dose-response curves, characterized by the asymptotic maximum mortality with increasing UV-B radiation and the UV-B radiation sufficient to achieve half of maximum mortality.

The experiments were conducted at the Obhur marine research station of King Abdulaziz University (Jeddah, Saudi Arabia, 21.71°N, 39.09°E) between June 1 and 7, 2013, the time of maximum solar and UV-B radiation in the region [21], [22]. Underwater UV-B penetration down to 3 m depth was measured using an underwater Solar Light UV-B sensor (model PMA 2104) at 0.5 m depth intervals at the center of the Obuhr Creek, the site of zooplankton sampling, at the mouth of the Creek and 1 Km offshore into the Red Sea. Incident underwater UV-B radiation, the most damaging component of the solar radiation spectra [6], just below the water surface was measured during the experiments at 5 min intervals using a logging underwater Solar Light UV-B sensor (model PMA 2104) placed at the aquaculture pond where the experiments were conducted.

Organisms were collected at dusk or dawn from coastal waters of the Red Sea off the KAU marine station at the Obhur Creek, about 12 Km long and 300 m wide, inlet, using shallow (top 10 m) vertical plankton tows with a WP2 net fitted with a 150 or 500 µm mesh, depending on taxa. The net was fitted with a large, 20 L, plastic bag to collect the animals, thereby avoiding pressure stress to the organisms. Immediately upon collection, bags with the organisms were placed in a cooling box with seawater, to maintain in situ temperature and transported in the dark to environmental chambers. In these experimental chambers, set at the in situ seawater temperature, which varied between 31°C and 34°C during the experimental period, the organisms were transferred to buckets and maintained in aerated filtered seawater in dim light. Between 50 and 100 individuals, depending on the taxa, were isolated using sterilized Pasteur pipettes with extreme care to avoid damages so that live intact animals (male and female) were sampled. A total of 100 healthy individuals, as assessed by their motion patterns, were isolated using sterilized Pasteur pipettes with extreme care to avoid damages so that live intact animals (male and female) were sampled. The number of *Labidocera* sp. individuals was only 50 because this taxa was never present in sufficient abundance to isolated a larger number of individuals (Table 1).

**Table 1:** Size and coloration of the zooplankton taxa tested and the mean (±SEM) experimentally determined maximum mortality (µ_max_) under UV-B radiation and excess mortality relative to that in the dark (µ_dark_) as well as the UV-B radiation, as percent of that just below the surface of the accumulated doses along the experiment, sufficient to raise mortality to 1/2 of µ_max_.

Species	Length (µm)	Colour	µ_max_ (h^−1^)	µ_dark_ (h^−1^)	µ_max_ (h^−1^)	UV-B_1/2__µmax_	
						%	KJ m^−2^
*Acartia* sp.	1286±35	Brown	0.052±0.015	0.017	0.035	1.5±1.8	0.65±0.078
*Lucifer* sp.	8008±212	Yellow-transparent	0.187±0.077	0.053	0.134	8.1±11.3	0.83±1.16
*Centropages* sp.	1692±42	Red	>0.031	0	>0.31	>50	>15.7
*Fenneropenaeus indicus*	222±3	White body	0.53±0.07	0.024	0.506	5.3±2.6	0.19±0.09
*Oncaea* sp.	599±49	Yellow-orange	0.083±0.02	0.024	0.059	10.7±8.2	4.65±3.58
*Macrostella* sp.	1224±41	Dark-orange	0.04±0.02	0.018	0.022	2.2±2.6	0.95±1.1
*Labidocera* sp.	2273±34	Black	>0.016±0.01	0.096	0.064	>12.0	>0.53
*Copilia* sp.	3901±70	Red-brown (female) Transparent (male)	0.11±0.02	0.049	0.061	5.1±4.3	0.27±0.23

The experiments were conducted with eight common zooplankton taxa, including six copepod taxa (*Acartia* sp., *Copilia* sp., *Centropages* sp., *Oncaea* sp., *Macrosetella* sp. and *Labidocera*sp.) and two decapods, *Lucifer* sp. and the nauplii of the Indian Ocean white prawn, *Fenneropenaeusindicus*, which was hatched at the aquaculture facility at the Obhur research station. For the first taxa tested, *Labidocera* sp., we used two duplicated quartz bottles containing 10 individuals each per treatment. However, we assessed that the experimental results were more robust if all 20 individuals were incubated in the same flask, so that all other taxa were tested by incubating 20 individuals contained in single quartz bottles for each treatment. Quartz flasks allowed the full spectrum of ambient solar radiation to penetrate into the bottles, across a range of solar radiation ranging from the full solar radiation incident below the water surface to darkness. The experiment involved, therefore, five light intensities: 100%, 21.6%, 7.2%, 3.2% and 0% of solar radiation. To produce these light levels quartz bottles were covered with multiple layers (0 through 3) of neutral screen to reduce the solar radiation incident on the bottles. The dark treatment, 0% of solar radiation, was achieved by enclosing the glass bottles in thick, dark plastic bags. All bottles were incubated, just under the water surface, submersed about 10 cm in an aquaculture pond at the research station, thereby maintaining the seawater temperature, which was recorded continuously using a calibrated HoBo light/temperature data logger, and fully exposed to the incoming solar radiation at 10 cm below the surface, thereby achieving the natural solar spectrum and photoperiod.

The experiments were initiated in the morning, typically around 8 am, and lasted, in general, less than 48 h, which sufficed to cause widespread mortality in the organisms receiving solar radiation. The number of dead organisms in each replicated bottle was counted at 4 to 6 h intervals during the daytime until mortality reached 90%. To do so, the experimental bottles were transported, inside a cooling box, into temperature-controlled chambers and the dead organisms counted, under dim light, as those that sank to the bottom and showed no motion. Upon counting, the bottles were returned to the experimental pond. Mortality rates (µ, units h^−1^), were then calculated as the slope of regression equation describing the decline in the natural log of the number of surviving organisms over time. Mortality rates (µ, units h^−1^) represent per capita rates and, therefore, represent the probability that anyone organism will die within one hour. If multiplied by 100, these represent mortality as % of individuals per hour. Accumulated UV-B doses were calculated by integrating the instantaneous values measured at 15 min intervals along the duration of the experiments. Where depletion of surviving organisms was steep, the mortality rates derived from the depletion curves contained uncertainty, as the logarithmic rates cannot be considered the sampling event when all organisms were dead and the 4 to 6 h interval introduced a limitation in the precision of the mortality rate estimated. However, this estimate could not be shortened without introducing artifacts derived from excess handling and disturbance of the organisms. Catastrophic mortality at 100% UV-B radiation incident below the surface for *Labidocera* precluded resolving µ_max_.

Dose-response curves were then constructed for each taxa tested as the relationship between the mortality rate (h^−1^) and the UV-B radiation, as percent of the incident radiation or the accumulated dose (KJ m^−2^), along the experiments was described using Michaelis-Menten equations of the form,



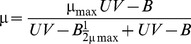
(1)


Where µ_max_ represents the asymptotic maximum mortality with increasing UV-B radiation and UV-B_1/2µmax_ represents the UV-B radiation sufficient to achieve half of maximum mortality (µ_max_).

## Results

The UV-B index at the surface during the experimental period averaged 11.9 (range 11.54 to 12.34) and the average (±SE) maximum daily values of the incident UV-B radiation just below the surface was 0.084±0.014 mW cm^−2^. The PAR levels incident on the experimental bottles was also high, reaching, on average, maximum values of about 1,340 µmol quanta m^−2^ s^−1^ and integrated daily values of about 8.8 quanta m^−2^. The water transparency increased rapidly offshore of the Creek, with the extinction coefficient for UV-B radiation at Obhur Creek, the site of zooplankton sampling, was 0.31±0.01 m^−1^, 0.28±0.01 m^−1^ at the mouth of the Creek, and 0.14±0.04 m^−1^ at 1 Km offshore, corresponding to depths of 10% of incident UV-B radiation of 7.4 m, 8.22 m, and 16.4 m, respectively. Accordingly, the radiation incident on the experimental quartz bottles receiving 3.2% of the incident radiation treatment tested (see Methods) was equivalent to that received in situ at 18.5 m, 20.5 m and 41 m depth at each of the three coastal locations, respectively.

**Figure 1:**
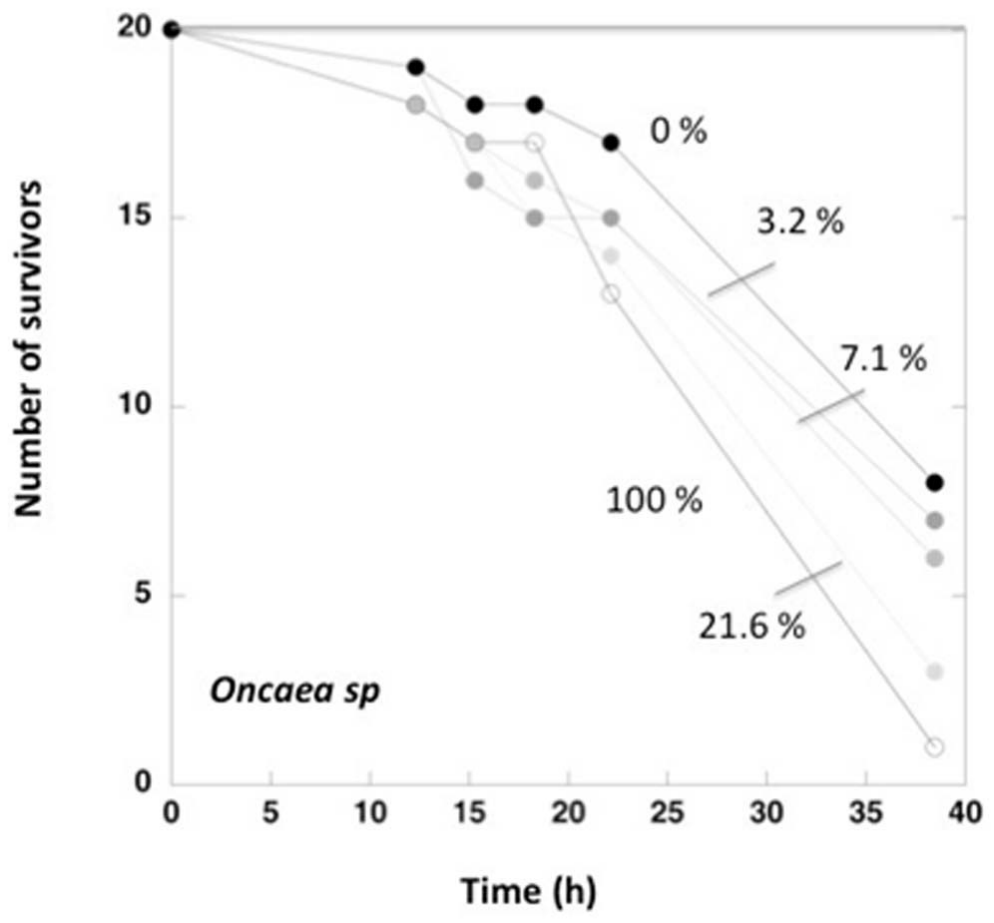
Sample depletion curves, for *Oncaea* sp., showing the decline in surviving individuals over time when experimentally exposed to different levels of UV-B radiation.

**Figure 2:**
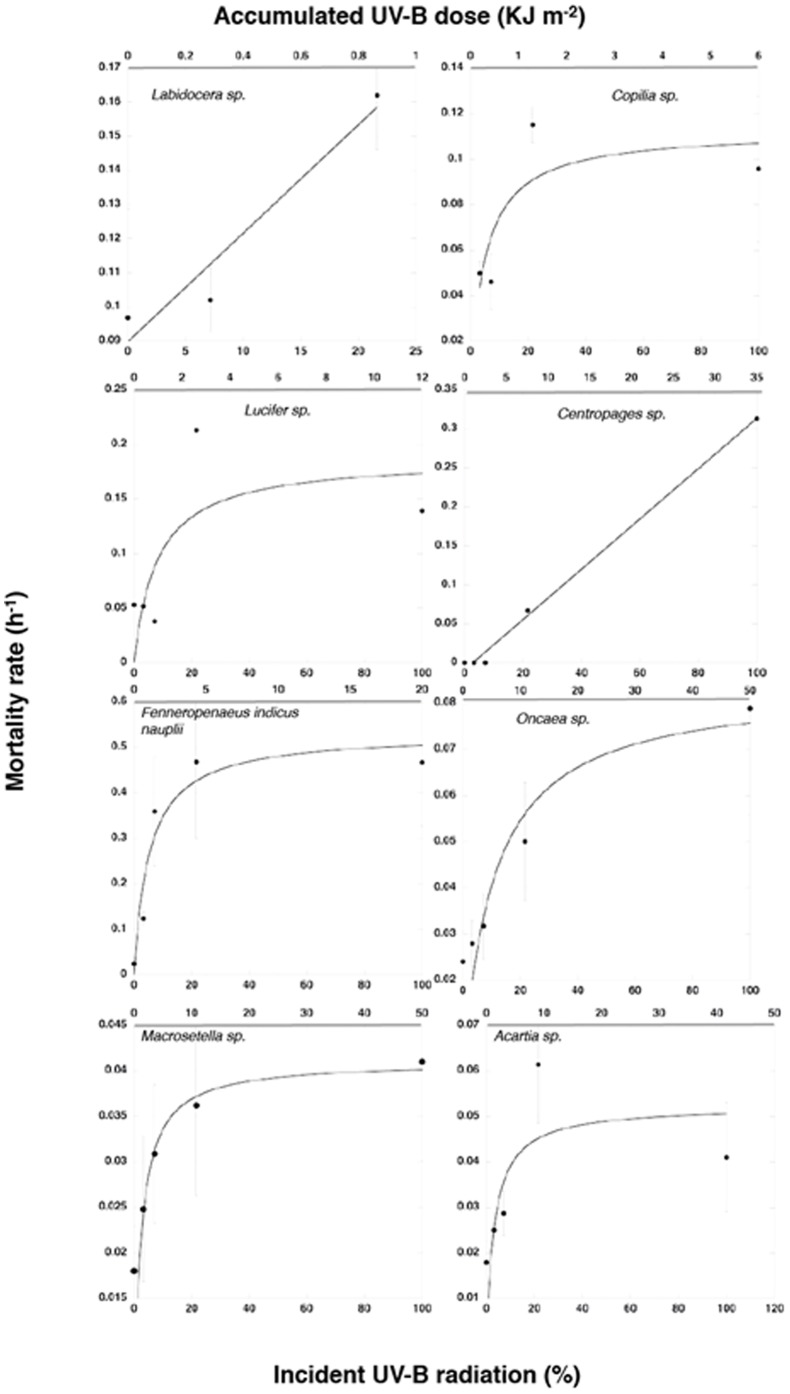
Dose-response curves describing the relationship between the mortality of zooplankton taxa and ambient UV-B levels (as % of UV-B incident below the surface or accumulated UV-B radiation along the experiments). The solid lines show the fitted linear regression of Michaelis-Menten equation (see Table 1 for parameters of the later). Catastrophic mortality at 100% UV-B radiation incident below the surface for *Labidocera* precluded resolving µ_max_.

The species tested ranged 40-fold in body length and differed in pigmentation, from rather transparent organisms, such as *Lucifer* sp. and *Copilia* sp., to dark, red-pigmented organisms, such as *Macrosetella* sp. (Table 1). The mortality under ambient temperature in the dark also ranged broadly, from negligible in *Centropages* sp. to as high as 0.11 h^−1^ in *Copilia*sp., with an average of 3.5±1.1% h^−1^ across taxa (median 2.4% h^−1^, Table 1). The number of surviving organisms declined over time, with the slowest decline in the dark and the steepest decline with increasing UV-B radiation received (Fig. 1). As a consequence, there were strong relationships between the mortality rate of the various species considered and the percent solar radiation, or the accumulated UV-B radiation, received over the experiment (Fig. 2). The shapes of the relationship between mortality rate and the percent UV-B irradiance or the accumulated UV-B radiation received different, however, among species (Fig. 2), with most species showing an asymptotic relationship between mortality rates and the percent solar radiation or the accumulated UV-B radiation along the experiment, which was best described by the Michaelis-Menten equation (Table 1), and the mortality rate of *Centropages* sp. showing a linear increase with UV-B over the range of UV-B radiation tested (Fig. 2).

**Figure 3:**
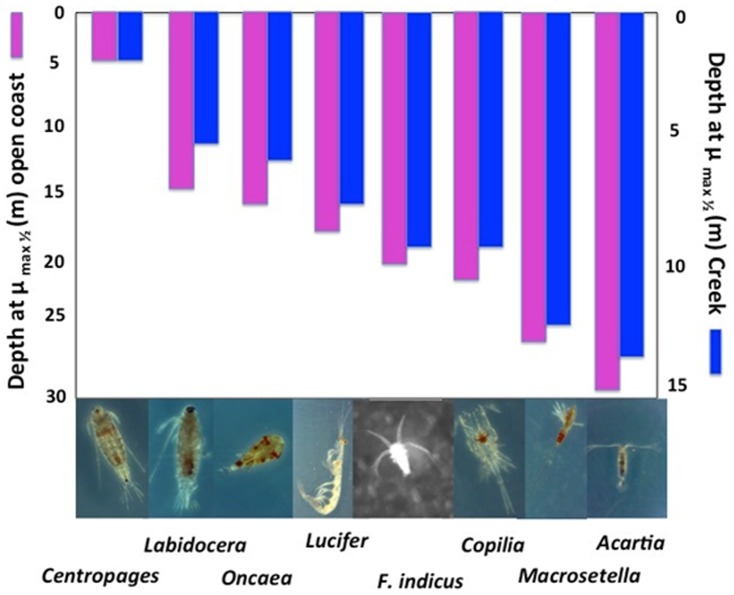
The depth at which sufficient UV-B radiation penetrates in the open coastal Red Sea waters (1 Km offshore from the Obhur Creek, magenta columns) and the Obhur Creek (blue columns) to raise mortality rates of different zooplankton taxa to 1/2 of µ_max_ (cf. **Table 1**).

The fitted Michaelis-Mensen equations allowed calculation of two key descriptors, the maximum mortality, µ_max_ (h^−1^), under the solar radiation incident underwater during the experiment, and the UV-B radiation at which mortality rates reached half of µ_max_, UV-B_1/2µmax_ (Table 1, Figs. 2 and 3). The µ_max_ averaged 18.4±5.8% h^−1^ across taxa (median 13.5% h^−1^), five-fold greater than the average mortality in the dark for the species assessed, and ranged almost 20-fold among taxa (Table 1), independently of their mortality rate in the dark (p>0.05). The nauplii of the prawn, *Fenneropenaeusindicus* exhibited the highest µ_max_ when exposed to ambient levels of solar radiation, while *Acartia* sp. and *Macrosetella* sp. exhibited the lowest µ_max_ (Table 1). The effect of solar radiation on mortality was represented by the excess µ_max_ relative to the mortality in the dark. Excess µ_max_ averaged 14.9±6.0% h^−1^ (median 6.25% h^−1^), only slightly below the mean µ_max_, indicating that most of the mortality experienced was attributable to solar radiation (Table 1).

UV-B_1/2µmax_ ranged from 1.5% to>50% of the UV-B radiation incident below the surface, averaging 12±5.6% of incident solar radiation across taxa (median 6.7%), corresponding to 12±5.6 KJ m^−2^ (median 0.74 KJ m^−2^, Table 1). This indicates, when combined with the measured light extinction coefficients for UV-B, that the solar radiation sufficient to raise mortality to half of µ_max_ radiation reaches down to depths ranging from 4.9 m to 29.9 m across taxa (mean 19.2±2.7 m) in clear coastal waters and 2.2 to 13.5 m (mean 8.6±1.2 m) in the more turbid waters of Obhur Creek (Fig. 3).

## Discussion

These results confirm that Red Sea zooplankton are highly vulnerable to ambient solar radiation, with preliminary experiments, removing UV-B radiation, demonstrating that mortality is induced by the UV-B component. Mortality rates increased steeply with moderate increases in UV-B radiation to reach maximum mortality levels at about 20% of the incident UV-B radiation below the surface. As a consequence, removing solar radiation reduced mortality rates down to, on average, 20.8% of mortality rates under maximum ambient solar radiation, much higher than the reduction in mortality rates to 60% under experimentally reduced UV-B radiation previously reported for crustaceans [6]. The much stronger mortality increase of the Red Sea zooplankton in responses to exposure to ambient solar radiation can be explained by the high UV-B doses underwater resulting from the combined high incident radiation and high water transparency allowing deep penetration of damaging UV-B radiation.

The experiments involved a limited number of individuals (N = 20) per treatment and, except for *Labidocera* sp. where duplicates were used (with N = 10), no replication within treatments, which represent a source of uncertainty and likely account for much of the variability in the derived dose-response parameters (Table 1). Whereas a larger number of organisms and replication would have possibly led to more robust results, this would have been possible only for the most abundant taxa. Use of different designs for different taxa would have added uncertainty in the comparisons among taxa, as the results for different species would have differed in power. On the other hand, use of a greater number of individuals per experiment, if possible across taxa, would have led to a longer time elapsed between collection and the onset of the experiment, as more time would have been required to sort the individuals, introducing additional artifacts, such as interference with the photoperiod and/or starvation and a general decline in condition of the organisms at the onset of the experiment.

Vulnerability to solar radiation varied widely across species, with *Centropages* sp. being the most resistant species to solar radiation and *Acartia* and *Macrosetella* being the most vulnerable ones, independently of size or pigmentation. Whereas relatively resistant species, such as *Centropages* sp., *Labidocera* sp., and *Oncaea*sp. tend to be dark colored, and transparent or white colored species, such as *F. indicus*, *Lucifer* sp. and *Copilia* sp., tend to be vulnerable, some colored species, such as *Acartia* sp. and *Macrosetella* sp. remained vulnerable. Strategies to increase zooplankton resistance to solar radiation include the accumulation of photoprotective pigments from ingested food [26] and the photoenzymatic repair of UV damages [27], [28]. However, accumulation of photoprotective pigments, often carotenoids, red colored, or mycosporin-like aminoacids, increase the detectability of the zooplankton to visual predators and may, therefore, carry negative impacts [29]. This may be particularly significant for relatively large taxa, such as *Lucifer* sp., which body remains, therefore, near transparent. Yet, the results presented here indicate that pigmentation and existing capabilities for photoenzymatic repair are insufficient to protect Red Sea zooplankton from damages due to ambient solar radiation levels, as mortality rates reached half of maximum levels at a median UV-B level of only 6.7% of that incident below the surface.

That even highly pigmented zooplankton taxa in the Red Sea community studied remain vulnerable to ambient levels of solar radiation suggests that zooplankton must remain at depth to avoid damaging UV-B doses, likely constraining their day-night migration behavior, as observed in alpine lakes [13], [30]. Specifically, our results suggest that zooplankton must remain below a median depth of 19 m in open coastal Red Sea waters to avoid UV-B exposure sufficient to raise mortality to 1/2 of µ_max_ (Fig. 3). This implies that only the most resistant among them can use the top half of the mixed layer during daylight, where most primary production occurs. The restriction on the food niche available to Red Sea zooplankton during daytime imposed by UV-B radiation may have important consequences in limiting the transference of production up the food web. In addition, changes in UV radiation, such as that found between more turbid coastal waters and more transparent open Red Sea waters, may lead to shifts in zooplankton communities by favoring more resistant taxa offshore.

These results are significant because they extend evidence of the significance of ambient UV-B levels as a stressor of zooplankton communities from high-mountain or high-latitude lakes, where most of the evidence of significant impacts of UV-B radiation originated [26], [29], [31], [32], to the oligotrophic ocean. Evidence of UV-B impacts on marine zooplankton are much fewer, limited to laboratory studies or studies in relatively turbid waters [15], [18]. Studies on the *Acartia* genus provided evidence of UV-B avoidance behavior in *Acartiahudsonica* [14] and *Acartiapacifica* [33] and demonstrated fitness costs of *Acartiatonsa* feed diets low in photoprotective pigments [17], and evidence that UV radiation reduces hatching success of the Arctic copepod *Calanusfinmarchicus* [20]. The analysis of dose-response levels of UV-B levels on a range of copepods in Puget Sound suggested they were resistant to ambient levels of UV-B radiation in those relatively turbid waters [18], similar to results from San Francisco Bay, where only 1% of UV-B radiation reaches to 0.5 m depth [15].

Hence, previous analyses of UV-B impacts on marine zooplankton communities suggested that these are relatively small or even negligible. However, these studies were conducted in relatively turbid waters, whereas the most vulnerable communities are expected to be those in tropical, oligotrophic waters where high incident solar radiation levels combine with extreme underwater penetration of UV-B radiation [11], [12], [34] to yield high UV-B doses. The steep increase in zooplankton mortality with ambient levels of solar radiation demonstrated here confirms the expectation that solar radiation is a significant stressor to zooplankton communities in oligotrophic waters. Because the oligotrophic ocean extends across 70% of the ocean surface, solar radiation can be a globally-significant stressor for the ocean ecosystem, by constraining zooplankton use of the upper levels of the water column and, therefore, the efficiency of food transfer up the food web in the oligotrophic ocean.

The results obtained here suggests that future efforts should focus on an improved understanding of the nature of the relationship between mortality rate and exposure to solar radiation, including examining the action spectra, i.e. the response to specific wavelengths of solar radiation [35], of mortality rates, and testing for the role of exposure time on mortality rates. The simple exponential model used here, the most parsimonious model provided the limited replication and the fact that survival was assessed at 4 h to 6 h intervals, assumes mortality rates to be constant, whereas other survival distributions, such as Weibull or Log linear, involve shifting mortality rates over time [36].

## Conclusions

The results presented here demonstrate that ambient solar radiation levels suffice to cause significant mortality on Red Sea zooplankton down to significant depths, in excess of 19 m, in the water column. Vulnerability to solar radiation varied widely across taxa, probably due to different repair mechanisms together with different pigmentation. The vulnerability of the Red Sea zooplankton community examined to solar radiation should lead to a severe restriction on the food niche available during daytime imposed by UV-B radiation, with important consequences in limiting the transference of production up the food web. As incident UV-B radiation has increased [7], [8] and the oligotrophic regions of the ocean maybe expanding [9]–[10], the results presented here suggest that UV-B radiation must be considered as a significant stressor on a key node of the ocean food web, likely affecting the transference of production up the food web in the oligotrophic ocean.
